# Resources consumption and environmental impacts of the DYNAMIC digital health intervention aimed at improving quality of care for sick children in Tanzania: a life cycle assessment

**DOI:** 10.3389/fdgth.2026.1788634

**Published:** 2026-05-28

**Authors:** Maxime Karlen, Nina Emery, Rainer Tan, Godfrey Kavishe, Peter Agrea, Sabine Renggli, Alexandra V. Kulinkina, Lameck Luwanda, Geofrey Isdory, Ibrahim Mtebene, Chacha Mangu, Pascale Schwab Castella, Xavier Bengoa, Valérie D’Acremont

**Affiliations:** 1 Centre for Primary Care and Public Health (Unisanté), University of Lausanne, Lausanne, Switzerland; 2 Ifakara Health Institute, Dar es Salaam, Tanzania; 3 Swiss Tropical and Public Health Institute, Allschwil, Switzerland; 4 University of Basel, Basel, Switzerland; 5 National Institute of Medical Research–Mbeya Medical Research Centre, Mbeya, Tanzania; 6 Faculty of Geosciences and Environment, University of Lausanne, Switzerland; 7 Adastra Sustainability, Choulex, Switzerland

**Keywords:** digital health sustainability, carbon footprint, life cycle assessment, resource consumption, planetary health

## Abstract

**Background:**

Health systems contribute to an important part of planetary boundaries overshoot, the effect of its rapid digitalization being however not well known. DYNAMIC is a Tanzanian digital health project, aimed at improving quality of care for sick children at primary care level through the provision of a tablet-based clinical decision support algorithm for clinicians. We evaluated the raw material resources and energy required by this intervention, as well as the environmental impacts, to inform strategies for improving its sustainability.

**Methods:**

Additional resources, including IT equipment, diagnostics and medicines used by the health intervention compared to usual care, were quantified. A life cycle assessment was conducted to calculate greenhouse gas emissions, fossil energy and mineral resources use, and damages to ecosystems and human health.

**Findings:**

GHG emissions of the intervention in 40 health facilities over one year, allowing to attend 90,992 children, were 20.6 tons of CO2-eq per year (PY). Medical supplies were the main source of emissions (13 tons), followed by digital supplies (5 tons), and logistics (2.6 tons). Fossil energy and mineral resources used were 380 GigaJoules and 77.9 kg deprived PY respectively. Damage on human health was 0.062 DALY, and on ecosystems 12,385 Potentially Disappeared Fraction of Species per m^2^ of land PY. The two-third decrease in antibiotic prescription as a result of the DYNAMIC project could reduce 14.5 tons of CO2-eq emissions.

**Interpretation:**

The digital component of the DYNAMIC health intervention increased its carbon footprint by a third, the main drivers remaining however the increase in medicines and medical devices use. Three quarters of the overall emissions could however be saved thanks to the antibiotic stewardship effect of the intervention. This shows that the rapid digitalization of health systems could accelerate their dependency on fossil fuels and other raw material. This negative effect on the environmental should be systematically evaluated to know if it is at least compensated by a benefit in terms of medical supplies savings. Promoting local, eco-friendly production of essential medical supplies and synergizing digital health interventions to use a shared IT infrastructure are also essential strategies for preserving resources and protecting the environment.

## Introduction

Digital health interventions are seen as a promising way to improve quality of care, especially in low-resource settings, because of their relatively low cost and scalability (1). On the other hand, the scarcity of raw materials (especially metals) and fossil fuels required to produce IT equipment, and the ecological footprint and electricity requirements of digital activities and infrastructure, are increasingly being recognized (2). Despite these concerns, resource requirements and environmental impacts of digital health initiatives are rarely considered. Existing literature primarily focuses on telemedicine in high income settings, and often reports a reduction in greenhouse gaz (GHG) emissions from patient travels reduction (3). However, these analyses tend to overlook the environmental burden of the digital infrastructure itself, including the extraction and processing of materials and the full lifecycle impacts of devices. Moreover, the other forms of environmental harm are rarely considered. On top emitting a lot of CO2 that accelerate climate change, the rapid digitalization of healthcare could significantly increase soil, air, and water pollution, and therefore contribute to environmental degradation and thus indirect deleterious human health effects (4–6). The rising incidence of natural disasters and their direct effects on human health is also well documented (5). Healthcare technologies, especially digital health interventions, could create a paradoxical situation where initiatives intended to improve quality of care, might in fact contribute to human health damages, while health systems are already grappling with the increasing burden of diseases linked to climate change, biodiversity loss and pollution (7, 8). Moreover, digital health programs could accelerate the depletion of energy sources and critical raw materials, including freshwater, many of which are becoming increasingly scarce at a global level.

**Figure 1:**
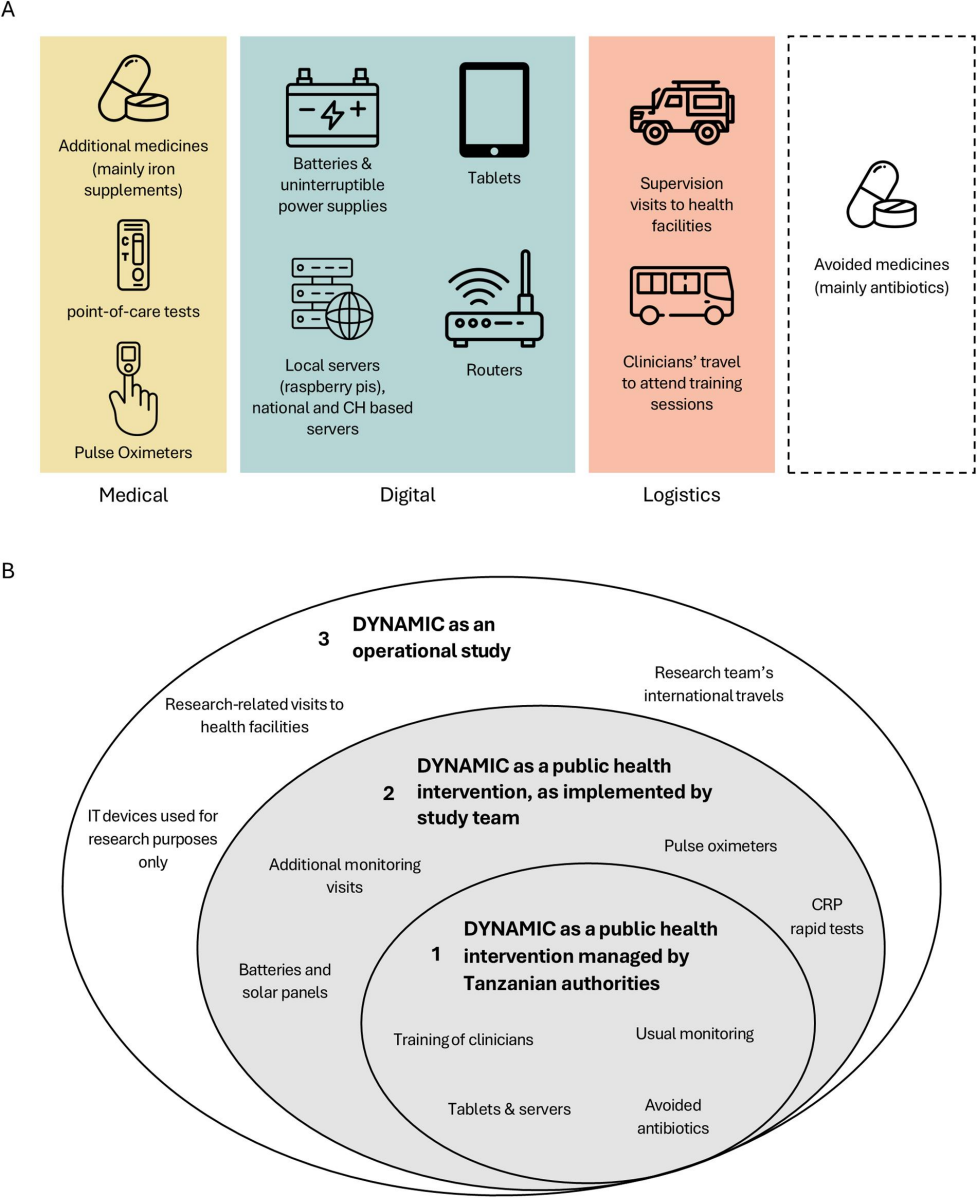
Scope of the analysis **(A)** DYNAMIC project implementation package. “Additional medicines” refer to treatments more frequently prescribed by clinicians when using the CDSA, while “avoided medicines” refer to those for which prescriptions were reduced—primarily antibiotics. **(B)** Illustration of different boundaries and examples of items included within each scope. The present analysis corresponds to scenario 2. Note that boundaries between the scenarios are partly subjective and that some items may span multiple scenarios.

To evaluate these various challenging issues associated with the digitalization of health system in practice, an evaluation of resource requirements and environmental impact was prospectively planned as part of the evaluation of the clinical impact of a digital health project called DYNAMIC. This project aimed to improve the management of sick children aged 0 to 15 years at the primary care level by providing clinicians with a tablet-based clinical decision support algorithm (CDSA). The intervention is described in detail in a previous publication (9). In summary, clinicians enter patients' symptoms and signs into the CDSA, which then suggests appropriate point-of-care tests and proposes a diagnosis based on the results. The clinical data collected through the tablets is first sent to a local server in the health facility and then to a central server based in the country. This data is used for clinician mentorship and monitoring of the project at the district level, as well as for disease surveillance and outbreak detection at the national level (9, 10). In addition to digital tools, essential diagnostic tests, usually not available at primary care level, were provided to support the intervention, such as pulse oximeters, hemoglobinometers, and rapid tests to measure C-reactive protein, a biomarker of possible bacterial infection (Figure 1). This intervention was thus influencing activities and infrastructure across multiple levels of the health system. Beyond the deployment of electronic and electrical equipment, it modified diagnostic and prescribing behaviors among clinicians, and reinforced supervision activities by district health management teams (Figure 1). The DYNAMIC project showed a nearly two-thirds reduction in antibiotic prescription thanks to more accurate diagnoses and better identification of children requiring antibiotic treatment (11, 12).

The objective of the present analysis was to measure the mineral and energy requirements and associated environmental impacts of the DYNAMIC digital health intervention over one year in 40 health facilities in Tanzania, and to assess the relative contribution of its digital and non-digital components in order to identify key drivers and inform strategies to improve its environmental sustainability. In addition to providing a bottom-up approach that can serve as a framework for further evaluation of the resource requirements and environmental impacts of digital health interventions, the aim was to allow national health authorities to take more informed decisions when scaling-up the intervention to additional districts.

## Methods

A Life Cycle Assessment (LCA) of the DYNAMIC health intervention in Tanzania was carried out to calculate the requirements in mineral and fossil resources, the greenhouse gas emissions (GHG), as well as the indirect effect and potential consequences on human health and ecosystems. Following standard LCA methodology, we did not include in the main calculation the consequences of the healthcare intervention, such as its effect on antibiotic prescription, since it may vary by context. However, we separately quantified the environmental benefit of antibiotics saving, and compared it to the overall environmental impact of intervention.

### Scope of the analysis

The functional unit was defined as one year of implementation of the DYNAMIC intervention. All necessary products and activities were assessed through their whole life cycle, from the extraction and processing of all raw materials used in purchases, through the end-of-life of all components. Capital goods (e.g., machinery and manufacturing equipment used in the production of finished goods) were included for production processes wherever data was available; they were however not included for distribution and retail stages. We considered that processes associated with the supply chain and waste management were taking place in Tanzania, although part of it might have taken place elsewhere in the world. Emissions potentially occurring beyond the reference year were not included.

At the time of data collection, a cluster-randomized controlled trial was ongoing, with half of the 40 health facilities receiving the intervention, and the other half assigned to a control group providing routine care. Because some activities involved both intervention and control health facilities (such as training or supervision), we considered that all health facilities were receiving the intervention (which happened for real at the end of the trial). We excluded activities and supplies exclusively related to research activities. However, we still included those required to enhance the health sector to be able to absorb this new healthcare intervention, acknowledging that health authorities might implement them in the future to support other interventions aimed at improving healthcare, beyond that proposed in the frame of the DYNAMIC project (Figure 1).

Any product and process related to the routine management of health facilities that were not modified by the DYNAMIC intervention, were considered out of scope (e.g., vaccine freezers, stethoscopes). Patients' activities happening outside the health facilities (e.g., purchase of non-prescribed medicines or travel to and from the health facility) were also excluded. Finally, in line with ISO 14044, when the necessary information was lacking and no reasonable estimate could be made, processes were excluded if their expected contributions to the total environmental impact was less than 1%.

### Life cycle inventory

An inventory of all products and processes was compiled using predefined inclusion and exclusion criteria (Supplementary Appendix S1) and reviewed by DYNAMIC study researchers based in both in Tanzania and Switzerland, whether directly involved in fieldwork or in coordination roles. For each material or energy flow quantified, the best available LCI dataset sourced from the *ecoinvent* database v3.8 was selected, using the cut-off by classification allocation model, with scaling factors applied when necessary (13). For example, in the absence of LCI data for fine chemicals such as active pharmaceutical substances (APIs), a scaling factor of 25 was applied to generic base chemicals from *ecoinvent*, based on one of the rare peer-reviewed LCA study on the impact of pharmaceuticals (14). For purchased products expected to have a lifespan longer than one year, allocation factors were applied (Supplementary Appendix S2, Supplementary Table S13). For example, laptops were expected to last 5 years, so their impact was divided by 5.

### Impact assessment

The IMPACT World + method was used for the impact assessment, focusing on a subset of indicators: climate change (midpoint), mineral resource use (midpoint), fossil energy use (midpoint), human health (damage) and ecosystems quality (damage) (15). The OpenLCA software was used to assist the LCA modelling and link the reference flows with the LCI database and link the LCI flows to the relevant characterization factors.

### Sensitivity analysis

Based on the situation prevailing during the project, we considered in a base scenario that all medicines (administered and avoided) were manufactured in India and transported directly to Tanzania by sea freight (5,500 km in total), while rapid tests were transported by air through commercial flights from India to Europe, and then from Europe to Tanzania (12,500 km in total). Given that the transportation of medical supplies accounted for a significant part of the total GHG emissions—and that future sourcing may vary with the availability of new suppliers—we also modelled a worst-case scenario in which all medicines were imported by commercial air freight from India via Europe.

### Ethics statement

This work was part of the DYNAMIC project (11), which obtained ethical approval in Tanzania from the Ifakara Health Institute (IHI/IRB/No: 11-2020), the Mbeya Medical Research Ethic Committee (SZEC-2439/R.A/V.1/65) and the National Institute for Medical Research Ethics Committee (NIMR/HQ/R.8a/Vol. IX/3486 and NIMR/HQ/R.8a/Vol. IX/3583), and in Switzerland from the cantonal ethics review board of Vaud (CER-VD 2020-02800). The present analysis used the data generated by the phase 1 study (a cluster-randomized controlled trial) of the DYNAMIC project (phase 2 consisting of the extension of the intervention to the control arm health facilities as well as new facilities). Written informed consent was obtained from all parents or guardians of participants when attending the participating health facility during the enrollment period. The first health facilities started enrolling patients on 01-12-2021, and the last health facilities started enrolling patients on 13-04-2022. Over 100 community engagement meetings with over 7,000 participants were conducted before and during the health intervention, including numerous meetings with Community and Regional Health Management Teams in the Mbeya and Morogoro regions of Tanzania.

## Results

For one year (November 2021 to October 2022) of operation of the DYNAMIC health intervention in 40 health facilities, allowing to treat 90,992 children, 49 tablets were distributed, and 42 routers, 40 raspberry pis and 23 UPS were installed. Clinicians performed an additional 76,424 point-of-care tests and prescribed an additional 183,002 tablets of medicines and 3639 tubes and bottles of medicines, while 754,324 tablets and 12,657 tubes and bottles of medicines were spared. 4,000 km were travelled for supervision and 8,000 km for training sessions. The summary of these main reference flows is listed in Table 1. The comprehensive life cycle inventory (LCI) is detailed in Supplementary Tables S1–S12, Supplementary Table S2.

**Table 1:** Main reference flows (non-exhaustive list) for one year of implementation of the DYNAMIC health intervention.

Category	Reference flow	Amount	Unit
Medical^a^	Additional medicines tablets per year—mainly iron supplements	183,002	Tablets
	Additional medicines bottles and tubes	3,639	Bottles & tubes
	Additional CRP point-of-care tests	20,610	Tests
	Additional Malaria point-of-care tests	6,624	Tests
	Additional Urine dipstick point-of-care tests	2,557	Tests
	Additional Hemoglobin point-of-care tests cuvettes	45,141	Tests
	Additional HIV point-of-care tests	1,492	Tests
	Additional pulse oximeters—expected life duration of 10 years	44	Pulse oximeters
Digital^b^	Local servers (raspberry pis)—expected life duration of 5 years	40	Servers
	Routers—expected life duration of 5 years	42	Routers
	Tablets—expected life duration of 3 years	49	Tablets (digital)
	Uninterruptible power supplies (UPS) and batteries—expected life duration of 5 years	23	Batteries
	Solar batteries and portable batteries—expected life duration of 10 years	14	Batteries
Logistics^c^	Distance travelled by car for supervision visits	4,000	Km
	Distance travelled by clinicians by bus for training sessions	8,000	Km
Avoided^d^	Avoided medicines tablets—mainly antibiotics	754,324	tablets
	Avoided medicines bottles and tubes—mainly cough syrups	12,657	Bottles & tubes

The expected life duration is specified for non-single use items, for which an allocation factor was applied to reflect their impact over one year.

a The full list of reference flows for added medicines is available in Supplementary Tables S1–S3, the full list of reference flows for added tests is available in Supplementary Tables S4, S5.

b The full list of reference flows in the digital category is available in Supplementary Tables S6–S9.

c The full list of reference flows in the logistics category is available in Supplementary Table S10.

d The full list of reference flows for avoided medicines is available in Supplementary Tables S1–S3.

**Figure 2:**
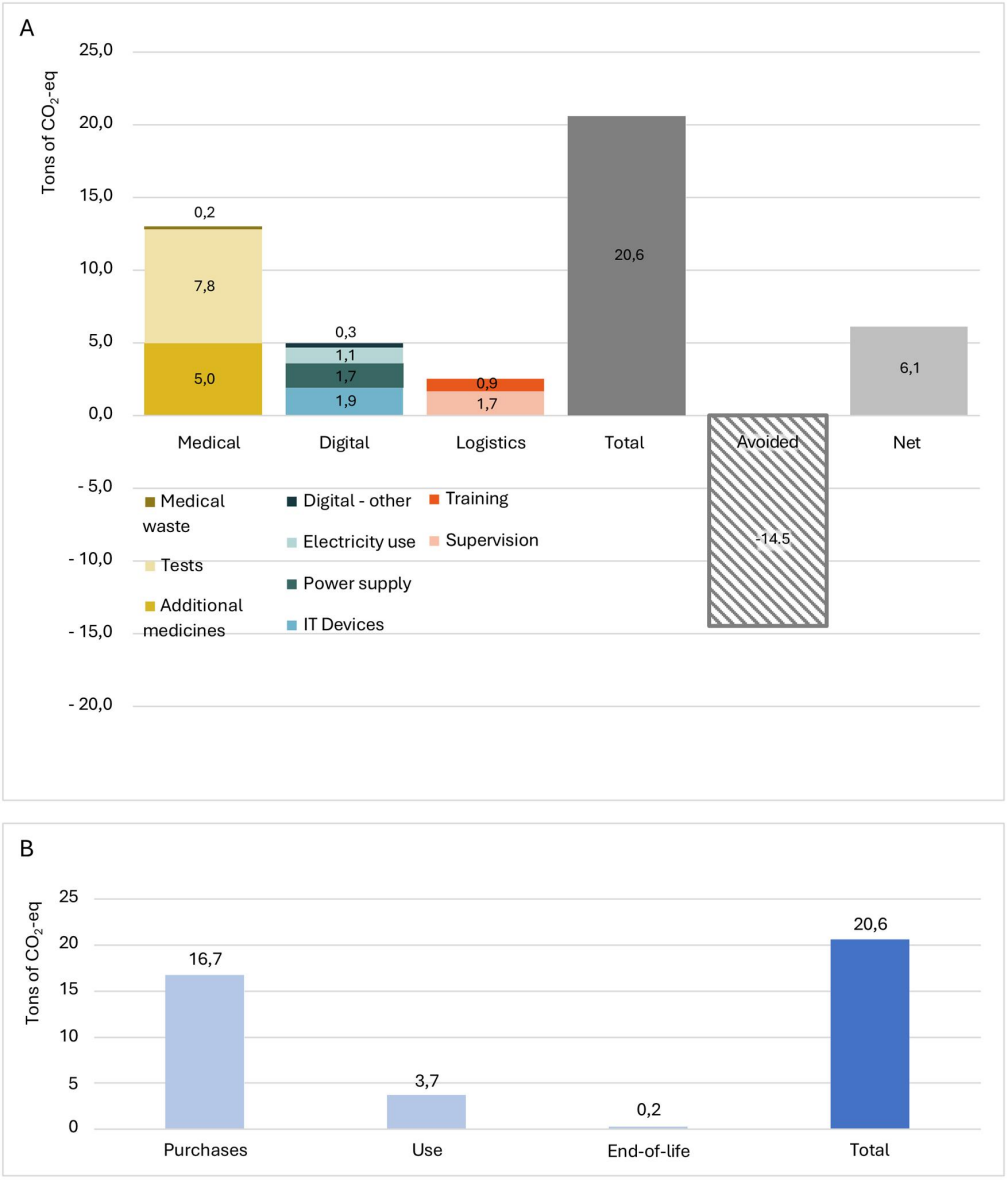
GHG emissions per year of implementation of the DYNAMIC intervention. **(A)** GHG emissions by category. **Medical**: “Medicines” and “Tests” account for the purchase of medicines and diagnostic tests, “Medical waste” accounts for the end-of-life stage of medicines and tests. There are no emissions related to the use stage of medicines and tests. **Digital**: “IT Devices” and “Power supply” account for the purchase of IT Devices (mainly tablets) and power supplies (mainly UPS and batteries), “Electricity use” accounts for the use stage of all electronic devices. The end-of-life stage of the digital category accounted for 0,002 ton of CO2-eq and is included in “Digital—other”. **Logistics**: “Supervision” accounts for all life cycle stages of supervision visits by car to health facilities, including purchase of cars and their end-of-life, “training” accounts for all life cycle stages of the transportation of clinicians by bus, to attend training sessions. The “net” impact is calculated as the total impact minus the impact of avoided medicines. **(B)** GHG emissions by life cycle stage. “Purchases” includes the extraction of raw material, production/manufacture and distribution. “Use” includes electricity use for digital devices and gas use for travel for supervision and training. “End of life” includes discharge of all products, whether recycled, burned or left in landfills.

The calculated GHG emissions were 20.6 tons CO2-eq per year. With 63% (13 tons CO2-eq) of all GHG emissions, medical supplies and activities accounted for the majority of emissions, followed by digital supplies and activities with 24% (5 tons CO2-eq) and logistics with 13% (2.6 tons CO2-eq) (Figure 2).

Among medical supplies and activities, diagnostic tests contributed to 60% (7.8 tons CO2-eq) of GHG emissions, mostly (37%; 4.8 tons CO2-eq) due to air transport, while additional medicines represented 38% (5 tons CO2-eq), with the production of APIs (30%; 3.9 tons CO2-eq) and of aluminum in packaging (6%; 0.8 tons CO2-eq) being the main drivers. Waste management of single-use medical equipment in Tanzania accounted for 1% (0.2 tons CO2-eq) of the impact of the medical category, 2/3 being caused by poorly managed waste paperboard from packaging (the only organic material likely to be degraded in unsanitary landfills or open dump sites).

Regarding digital supplies and activities, the purchase of IT devices, such as tablets, routers and laptops, represented 38% (1.9 tons CO2-eq) of emissions due to this category. The purchase of equipment necessary to ensure power supply (batteries and uninterruptible power supply (UPS), used when the main power input fails, generated 34% (1.7 tons CO2-eq) of these emissions. The electricity required to operate all these electronic devices represented 22% (1.1 tons CO2-eq) of the emissions, while 6% (0.3 ton CO2-eq) were due to the purchase of steel security boxes used to store valuable IT devices in health facilities.

Regarding logistics, emissions were due to road transportation, 66% (1.7 tons CO2-eq) related to supervision visits to health facilities and 34% (0.9 tons CO2-eq) for training sessions of clinicians.

As the DYNAMIC intervention allowed to reduce the medicine prescriptions (mainly antibiotics) by two-thirds, 14.5 tons CO2-eq could be saved, bringing the net carbon footprint of the project over one year to 6.1 tons CO2-eq.

**Figure 3:**
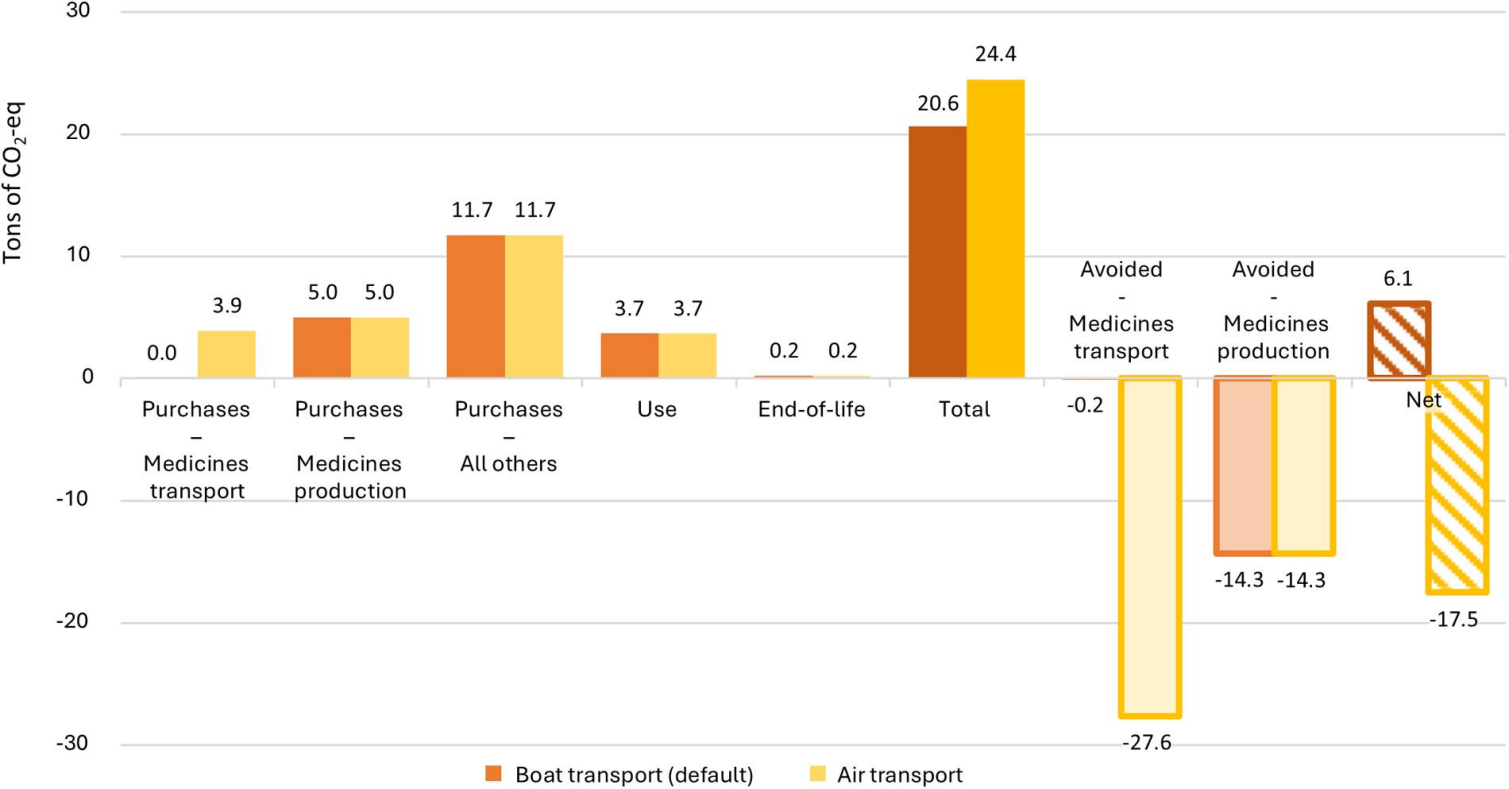
GHG emissions of the DYNAMIC intervention per year, under two scenarios for the transportation of used and avoided medicines (by sea or air freight). The “Purchases” category is divided into “medicines transport”, “medicine production”, and “all others” which entails the extraction of raw material, production and distribution of all other items. The avoided emissions are divided into “Avoided—medicines transport” and “Avoided—Medicines production”. The avoided emissions due to the end-of-life stage are negligible and not represented.

The results of the sensitivity analysis on the mode of transport of medicines are detailed in Figure 3.

The total GHG emissions of the DYNAMIC health intervention were moderately sensitive to the mode of transport of medicines, with a 18% increase if they had been all transported by air freight. However, avoided emissions from reduced medicine prescriptions were highly sensitive to transport mode, reaching 27.6 tons CO₂-eq under the air freight scenario. As a net result, if medicines were transported by air rather than sea freight, the benefits from avoided medicines would allow to entirely offset the GHG emissions due to the implementation of the health intervention itself.

**Figure 4:**
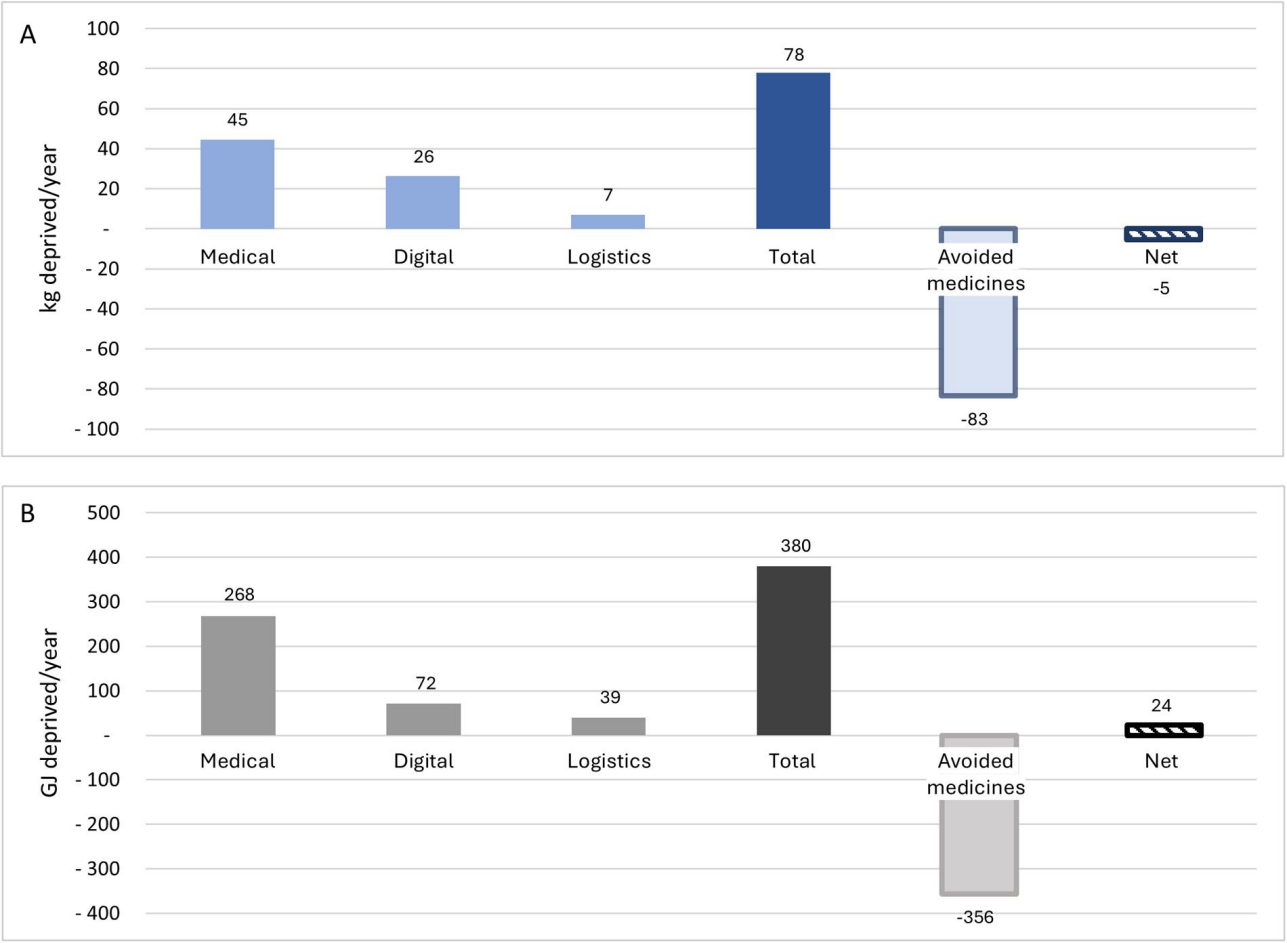
**(A)** impact on mineral resources use broken down by category. The main mineral resources depleted were clay, rare earths (tantalum) and bentonite. **(B)** Impact on fossil energy use broken down by category. The main fossil resources depleted were crude oil (refined into jet fuel, diesel and ethylene) and hard coal (for electricity production).

**Figure 5:**
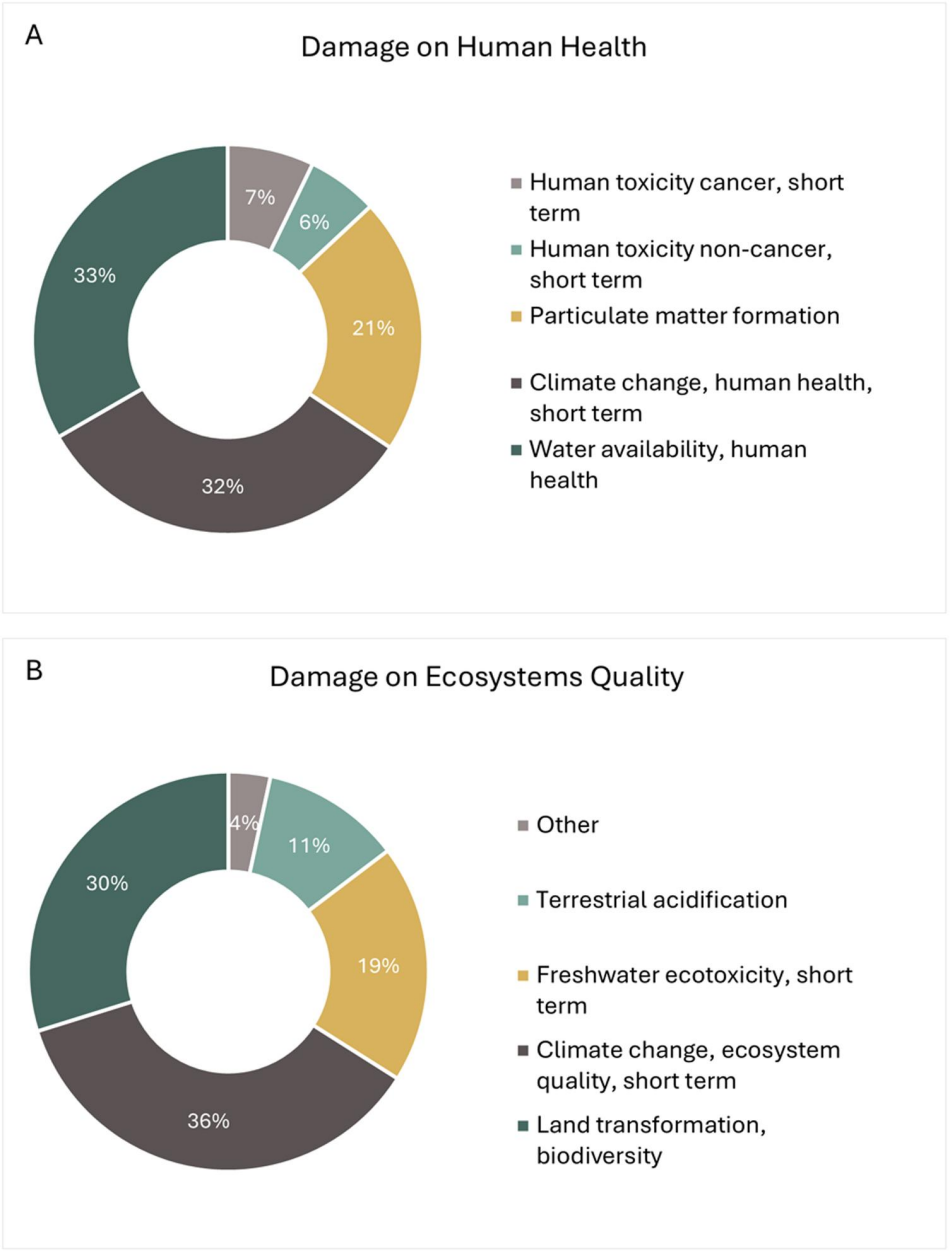
**(A)** damage on human health broken down by midpoint impact categories. This metric represents the diffuse risk for humans of being exposed to air and water pollutants, increasing scarcity of abiotic resources and climate change throughout the complete life cycle of a given human activity. It is not a measure of acute or chronic exposure to a specific disturbance such as a toxic substance, a pathogen or a local extreme weather event. **(B)** Damage on ecosystems quality broken down by midpoint impact categories. This metric is a representation of the combined pressure on ecosystems from excess nutrients (phosphorus and nitrogen) and toxic chemicals discharged in the environment, climate change, water scarcity, as well as land occupation and land transformation patterns.

The calculated mineral resource and fossil energy required to implement the DYNAMIC health intervention were 78 kg and 380 GJ (or 105,555 kW/H) per year respectively (Figure 4). The contribution of electric and electronic devices to the impact on mineral resource use (33%) was almost double that to the impact on fossil energy use (19%). The benefits from avoided medicines were larger on fossil energy use (94% offset) than CO2 emissions (70% offset). The calculated damage on human health and ecosystems quality was 0.062 DALY and 11,979 PDF*m^2^ per year respectively (Figure 5). Damage on human health was primarily driven by water availability (33%) and climate change (32%). The damage on ecosystems quality was driven primarily by climate change (36%) and land transformation (30%).

## Discussion

To the best of our knowledge, this is the first published LCA of a digital health intervention conducted in a sub-Saharan country (16). We found that the implementation of the DYNAMIC project in 40 primary care facilities in Tanzania generated close to 21 tons of CO_2_ equivalents over one year. Digital supplies and activities increased the impact of the intervention by a third. Medical supplies and activities (mainly medicine and diagnostic tests) were still the main sources of environmental damages, accounting for more than half of the overall emissions. Logistics (mainly transportation by car of district and health facility personnel for training and supervision) had also a significant impact.

Regarding medical supplies and activities, the main sources of emissions were caused by diagnostic tests, followed by medicines. Combined, these two elements represented 62% (12.8 tons) of all GHG emissions. The main source for the tests was the air transport from India to Tanzania. This demonstrates the importance of avoiding air freight as much as possible, for example by favoring sea freight or, even better, by developing local production. The COVID-19 pandemic illustrated that setting up an indigenous production of medical supplies in Africa is challenging—although the manpower and expertise are there—due to medical resources monopoly by the most geopolitically powerful countries (17) as well as economic constraints related to investment requirements, production costs, and reliance on imported inputs. As seen with some countries in South America and Asia, it is essential to foster local pharmaceutical and medical tests productions in low- and middle-income countries to improve access to medicines and diagnostic tests, promote knowledge transfer, strengthen economic sustainability and lower the carbon footprint (18).

The significant impact associated with medicines was mainly due to the production of active pharmaceutical ingredients (APIs), which is consistent with findings from other studies (14, 19, 20). Because mitigating the impact of API production might be difficult, reducing unnecessary medicine prescriptions is also key, and not only beneficial for human health, but also for the environment. This is well highlighted by the effect of avoided antibiotic prescriptions in the DYNAMIC project, that allowed to prevent almost three quarters of the emissions due to the intervention. Would these medicines be transported by air freight, the benefit would be even greater, with all emissions being compensated.

The purchase of IT devices (especially tablets) and electricity use also represented a consequent part of the emissions, and an even greater part of the mineral resource use. As a solution, the algorithm could be adapted for the use on computers (rather than tablets), which have recently been deployed in some health facilities and have a longer lifespan. It also highlights the need for good coordination between digital health interventions, to avoid duplication of electronic devices. Supporting local communities in getting access to renewable electricity will also help to keep the project's carbon footprint as low as possible while ensuring power autonomy in regions that are regularly affected by power outages.

To better understand the implication of the environmental impact generated by the DYNAMIC health intervention, it is helpful to use benchmarks. The 20.6 tons CO2-eq emitted annually by the DYNAMIC intervention represent the emissions of about 69 Tanzanians, or approximately 2 Swiss citizens in 2020, with 0.3 tons CO2-eq per year emitted for a Tanzanian that year, vs. 12.4 tons CO2-eq for a Swiss (national emissions adjusted for trade), according to Our World in Data (21). Although we did not assess the emissions associated with the overall routine operation of a primary health facility in Tanzania—and, to our knowledge, no such study exists—a relevant comparison can be drawn from Switzerland, where the average primary care practice produces approximately 30 tons of CO₂-eq per year. This is 58 times more than the emissions associated with the DYNAMIC intervention per Tanzanian facility.

The 380 GJ of primary energy used are equal to approximately 64 barrels of oil equivalent, which represents the annual oil consumption of 197 Tanzanians or 9 Swiss in 2018, according to the International Energy Agency. The 77.9 kg of mineral resources used yearly by the DYNAMIC intervention represents 9% of the annual consumption of one East African citizen, or 0.9% of one European citizen in 2017 (data by country are not available) (22, 23). For the calculated potential damage on human health and ecosystems quality, it is difficult to benchmark the absolute results, however the analysis gives a reasonable direction of potential hotspots (main impact through climate change, water availability and land transformation).

The numbers exposed in the above comparison highlight the inequities between low and high-income countries regarding the present climate emergency (24, 25). In a country where access to health care and medical technology is limited, the environmental cost of a digital health intervention like DYNAMIC may be outweighed by its clinical and public health benefits. For example, the intervention allowed to prevent the consumption of two thirds of antibiotics per year compared to routine care, which is essential considering the burden that antimicrobials resistance will represent in the future (26).

That said, we can also argue that in the current climate emergency, every additional kilogram of CO2 in the atmosphere counts and should be avoided, as well as every additional damage to ecosystems, regardless of the context in which a project is implemented. Public health interventions are no exception. Moreover, health systems share a significant amount of the global damage inflicted on the environment. Studies show that the carbon footprints of the health sector account for 3% to 10% of the national or global CO2 footprint (19, 27, 28). Recent research from Andrieu et al. looked at the relationship between healthcare systems’ resource footprints and their access and quality (29). For the 49 regions under review, Switzerland's health system has one of the best healthcare access and quality (HAQ) index but the worst energy footprint per capita. Spain's or Sweden's health system, while having an HAQ index similar to that of Switzerland, uses about four and three times less energy respectively. African health systems have a very low energy impact, but a HAQ index that is also well below Western health systems. In a best-case scenario, African countries will have to continue to improve their healthcare access and quality, while avoiding the inefficient and unsustainable resource-use patterns seen in high-income countries. Healthcare being an incompressible need, analyses such as the present LCA help to evaluate if a healthcare program or project is sustainable and worth supporting in a resource-limited world. While African health systems develop and improve, knowing the most ecological healthcare solutions will help make sustainable decisions and becoming more resilient to future resources scarcity and shortages. Indeed, as described by Zywert and Quilley, we are witnessing a growing vulnerability of health systems that depend on welfare states and continued economic growth, despite clear ecological limits to that growth and upcoming economic recessions (7, 30). It pushes us to act and redefine health systems that embeds the respect of our environment and resilience at its core.

This study has several limitations. First, our findings should be interpreted with caution given the uncertainties in the underlying data. Limited data availability, notably the absence of probability distributions for key reference flows, precluded a formal quantification of uncertainty such as Monte Carlo simulation. The main sources of uncertainty are described below, relating to the production of API, transportation of medicines, waste management, and applicability of European LCI datasets to African or Asian manufacturing contexts.

Regarding GHG emissions associated with API production, the same ecoinvent variable was used for all API (chemical organics with a scaling factor of 25, based on Wernet et al.). However, a recent study suggests substantial variations in impact depending on the complexity of API synthesis, and possible underestimation of carbon footprint by prior studies. This could affect the impact of both added and avoided medicines in our analysis. Regarding transport, the mode of transportation of medicines had a moderate to high influence on the results and is highly dependent on contextual factors such as future supplier availability or structural changes in the supply chain. Regarding waste, assumptions made on management schemes for plastic, aluminium, glass and electronic equipment in the Tanzanian context remain highly uncertain. When not burned, those materials are not major sources of GHG but their mismanagement can cause multiple other impacts on the environment and human health, primarily through the release of toxic substances into the soil, which can later contaminate surface and groundwater. Such pollution is poorly captured by LCA unless dedicated measurements on the chemical composition of waste streams are made. Finally, many LCI datasets used in the model are based on European operations and may not accurately reflect manufacturing practices in African or Asian countries (e.g., for medicines and chemicals manufactured in India).

Several other limitations relate to the scope of our analysis. First, methods such as IMPACT World + characterize the most known pollutants to provide the best estimate of environmental impact but cannot characterize the complete array of emissions released to soil, air and water. Second, we did not evaluate the total emissions associated with a regular health facility in Tanzania. It was thus impossible to estimate the proportion of environmental damage associated with the DYNAMIC digital health intervention as compared to the emissions already generated by routine care. Third, this analysis did not account for the effects of antimicrobial resistance (AMR), which is a major environmental and health consequence of antibiotic use.

Additionally, it should be noted that the majority of the authors of the present study was also involved in the DYNAMIC project, which could in principle introduce bias toward favorable results and lower environmental impact. To mitigate this, data collection was supervised by- and analysis was performed by an environmental engineer (XB) external to the project, and all methodological choices are fully documented in the article and appendices, mitigating the risk of undisclosed bias in the analysis.

Finally, our findings reflect the specific characteristics of the DYNAMIC project, and should be interpreted accordingly. Generalization to other digital health interventions involving different tools, medical products, or logistical processes requires caution.

In conclusion, this analysis highlights the importance of assessing the environmental impacts of health interventions, especially based on digital tools. Indeed, the IT component of the digital health intervention we analyzed here increased the carbon footprint impact by a third, highlighting the serious risks to the environment—and of high dependency on energy sources and raw material, including water—of the rapid and extensive digitalization of health systems taking place at present. If we had included in our analysis the impact of statistical analyses of the data generated by the project, the impact would have been probably larger, especially when using machine-learning.

Our results can guide decision making for the next steps of the deployment of the DYNAMIC intervention,. The DYNAMIC intervention would generate around 4,200 tons of CO2-eq per year if it would be extended to the 8,145 health facilities inventoried in Tanzania in 2021 (which remains little compared to the 18 million tons emitted by the country in 2020).

The digital component is a major driver of added environmental impact, and its contribution should be carefully optimized relative to the medical benefits delivered. Scale-up is most justified in settings where demonstrable health gains clearly outweigh the additional environmental footprint. All projects implementing new digital health systems should take environmental aspects into account, in addition to medical and economic considerations. Life cycle assessments (LCAs) are a useful tool for identifying the main environmental impact drivers of a project, and both researchers, implementers, and policymakers should consider them as part of their overall evaluation and decision-making process. As for all other sectors of our society, the environmental impact of any health intervention should be considered and weighed against its health benefits and overall utility, while also taking into account the principle of climate equity between Southern and Northern countries.

## Data Availability

The original contributions presented in the study are included in the article and in Supplementary Material. Further inquiries can be directed to the corresponding author.

## References

[1] Skolnik RL. Mhealth: using mobile technology to improve the health of the poor in poor countries. In: Riegelman RK, editor. Global Health 101. 3rd ed. Burlington, MA: Jones & Bartlett Learning (2016). p. 480–81.

[2] Belkhir L Elmeligi A. Assessing ICT global emissions footprint: trends to 2040 & recommendations. J Clean Prod. (2018) 177:448–63. 10.1016/j.jclepro.2017.12.239

[3] Lokmic-Tomkins Z Davies S Block LJ Cochrane L Dorin A von Gerich H. Assessing the carbon footprint of digital health interventions: a scoping review. J Am Med Inform Assoc JAMIA. (2022) 29(12):2128–39. 10.1093/jamia/ocac196 36314391 PMC9667173

[4] Thompson M. The environmentally impacts of digital health. Digit Health. (2021) 7:20552076211033421. 10.1177/20552076211033421 34408902 PMC8365173

[5] Romanello M Napoli CD Drummond P Green C Kennard H Lampard P. The 2022 report of the lancet countdown on health and climate change: health at the mercy of fossil fuels. Lancet. (2022) 400(10363):1619–54. 10.1016/S0140-6736(22)01540-9 36306815 PMC7616806

[6] Dominski FH Lorenzetti Branco JH Buonanno G Stabile L da Silva MG Andrade A. Effects of air pollution on health: a mapping review of systematic reviews and meta-analyses. Environ Res. (2021) 201:111487. 10.1016/j.envres.2021.111487 34116013

[7] Zywert K Quilley S. Health systems in an era of biophysical limits: the wicked dilemmas of modernity. Soc Theory Health. (2018) 16(2):188–207. 10.1057/s41285-017-0051-4

[8] Lawler OK Allan HL Baxter PWJ Castagnino R Tor MC Dann LE. The COVID-19 pandemic is intricately linked to biodiversity loss and ecosystem health. Lancet Planet Health. (2021) 5(11):e840–50. 10.1016/S2542-5196(21)00258-8 34774124 PMC8580505

[9] Tan R Cobuccio L Beynon F Levine GA Vaezipour N Luwanda LB. ePOCT+ and the medAL- suite: development of an electronic clinical decision support algorithm and digital platform for pediatric outpatients in low- and middle-income countries. PLOS Digit Health. (2023) 2(1):e0000170. 10.1371/journal.pdig.0000170 36812607 PMC9931356

[10] Cobuccio LG Faivre V Tan R Vonlanthen A Beynon F Barchichat E. medAL-suite: a software solution for creating and deploying complex clinical decision support algorithms. BMC Med Inform Decis Mak. (2025) 25(1):249. 10.1186/s12911-025-03077-6 40615862 PMC12226914

[11] Tan R Kavishe G Luwanda LB Kulinkina AV Renggli S Mangu C. A digital health algorithm to guide antibiotic prescription in pediatric outpatient care: a cluster randomized controlled trial. Nat Med. (2024) 30(1):76–84. 10.1038/s41591-023-02633-9 38110580 PMC10803249

[12] Tan R Kavishe G Kulinkina AV Renggli S Luwanda LB Mangu C. Quality of care when using a digital clinical decision support algorithm to manage sick children at primary care health facilities in Tanzania: a cross-sectional cluster randomized controlled trial (DYNAMIC study) [Internet]. medRxiv. p. 2024.04.05.24305394 (2024). Available online at: https://www.medrxiv.org/content/10.1101/2024.04.05.24305394v1 (Accessed 2024 October 17)

[13] Weidema B Bauer C Hischier R Mutel C Nemecek T Reinhard J. Overview and methodology. Data quality guideline for the ecoinvent database version 3 [Internet]. St. Gallen: The ecoinvent Centre; 2013 May. Report No.: 1(v3) (2013). Available online at: https://lca-net.com/files/Overview_and_methodology.pdf

[14] Wernet G Conradt S Isenring HP Jiménez-González C Hungerbühler K. Life cycle assessment of fine chemical production: a case study of pharmaceutical synthesis. Int J Life Cycle Assess. (2010) 15(3):294–303. 10.1007/s11367-010-0151-z

[15] Bulle C Margni M Patouillard L Boulay AM Bourgault G De Bruille V. IMPACT World+: a globally regionalized life cycle impact assessment method. Int J Life Cycle Assess. (2019) 24(9):1653–74. 10.1007/s11367-019-01583-0 PMC7592718 33122880

[16] Drew J Christie SD Rainham D Rizan C. HealthcareLCA: an open-access living database of health- care environmental impact assessments. Lancet Planet Health. (2022) 6(12):e1000–12. 10.1016/S2542-5196(22)00257-1 36495883

[17] Kavanagh MM Erondu NA Tomori O Dzau VJ Okiro EA Maleche A. Access to lifesaving medical resources for African countries: cOVID-19 testing and response, ethics, and politics. Lancet. (2020) 395(10238):1735–8. 10.1016/S0140-6736(20)31093-X 32386564 PMC7252104

[18] da Fonseca EM. How can a policy foster local pharmaceutical production and still protect public health? Lessons from the health-industry complex in Brazil. Glob Public Health. (2018) 13(4):489–502. 10.1080/17441692.2017.1396354 29098942

[19] The Shift Project. Décarbonner la santé pour soigner durablement [Internet] p. 155 (2021). Available online at: https://theshiftproject.org/wp-content/uploads/2021/11/211125-TSP-PTEF-Rapport-final- Sante.pdf (Accessed August 12, 2022).

[20] Siegert MW Saling P Mielke P Czechmann C Emara Y Finkbeiner M. Cradle-to-grave life cycle assessment of an ibuprofen analgesic. Sustain Chem Pharm. (2020) 18:100329. 10.1016/j.scp.2020.100329

[21] Our World in Data [Internet]. Per capita consumption-based CO₂ emissions. Available online at: https://ourworldindata.org/grapher/consumption-co2-per-capita (Accessed June 21, 2022).

[22] Baninla Y Lu Y Zhang Q Omotehinse AO Zheng X Zhang M. Material use and resource efficiency of African sub-regions. J Clean Prod. (2020) 247:119092. 10.1016/j.jclepro.2019.119092

[23] Eurostat [Internet]. Raw material consumption (RMC) by main material categories EU 2000-2020 (tons per capita). Available online at: https://ec.europa.eu/eurostat/statistics-explained/index.php?title=Material_flow_accounts_statistics_-_material_footprints (Accessed July 5, 2023).

[24] Marcantonio R Javeline D Field S Fuentes A. Global distribution and coincidence of pollution, climate impacts, and health risk in the anthropocene. PLoS One. (2021) 16(7):e0254060. 10.1371/journal.pone.0254060 34288922 PMC8294505

[25] Zhuang S Bolte G Lakes T. Exploring environmental health inequalities: a scientometric analysis of global research trends (1970-2020). Int J Environ Res Public Health. (2022) 19(12):7394. 10.3390/ijerph19127394 35742642 PMC9223819

[26] Lambraki IA Cousins M Graells T Léger A Abdelrahman S Desbois AP. Governing antimicrobial resistance (AMR) in a changing climate: a participatory scenario planning approach applied to Sweden in 2050. Front Public Health. (2022) 10:831097. 10.3389/fpubh.2022.831097 35874997 PMC9298947

[27] Lenzen M Malik A Li M Fry J Weisz H Pichler PP. The environmental footprint of health care: a global assessment. Lancet Planet Health. (2020) 4(7):e271–9. 10.1016/S2542-5196(20)30121-2 32681898

[28] Pichler PP Jaccard IS Weisz U Weisz H. International comparison of health care carbon footprints. Environ Res Lett. (2019) 14(6):064004. 10.1088/1748-9326/ab19e1

[29] Andrieu B Marrauld L Vidal O Egnell M Boyer L Fond G. Health-care systems' resource footprints and their access and quality in 49 regions between 1995 and 2015: an input-output analysis. Lancet Planet Health. (2023) 7(9):e747–e758. 10.1016/S2542-5196(23)00169-9 37673545 PMC10495829

[30] Zywert K. Human health and social-ecological systems change: rethinking health in the anthropocene. Anthr Rev. (2017) 4(3):216–38.

