# Supplementary material for: Resources consumption and environmental impacts of the DYNAMIC digital health intervention aimed at improving quality of care for sick children in Tanzania: a life cycle assessment
Source: Front Digit Health. 2026 May 28;8:1788634. doi: 10.3389/fdgth.2026.1788634 (PMC13254170; doi:10.3389/fdgth.2026.1788634)
Supplement: Supplementary file 1 [file Datasheet1.docx]

## S1 Appendix: Inclusion/exclusion criteria of products and processes in life cycle inventory

### **Inclusion criteria**

1. The activity or supply is related (even partially) to the basic functioning of ePOCT+ (the clinical decision support algorithm implemented by the DYNAMIC project) :
   1. E.g., tablets used by clinicians, oximeters and CRP tests, extra training attended by clinicians to learn how to use the tool: those are essential to the basic functioning of ePOCT+ as it is implemented today.
2. If an item is not exclusively related to the basic functioning of ePOCT+ but also has other purposes (ex. added solar panels are also used for the health facility lights), it shall still be included, and its allocation key adapted to take into account those other purposes.
3. The activity or supply was added (or avoided) because of ePOCT+ implementation.
   1. Examples of added items : UPS and batteries, medical devices, in particular diagnosis tests, and medicine prescriptions added compared to routine care, added supervision visits.
   2. Examples of avoided items : cough syrups and antibiotic prescriptions avoided compared to routine care.

### **Exclusion criteria**

1. The activity or supply is related exclusively to research activities related to the ePOCT+ implementation, e.g., tablets used by the research assistants, QR-code printers used to label informed consent forms.
2. The activity or supply is present in the routine health facilities’ activities and is neither increased nor decreased as the result of ePOCT+ implementation, e.g., blood centrifuges, electricity consumption of vaccine freezers.
3. Patients’ activities outside of the health facilities, e.g., patients commuting to the health facilities, hospitalisations, non-prescribed
