# Supplementary material for: Resources consumption and environmental impacts of the DYNAMIC digital health intervention aimed at improving quality of care for sick children in Tanzania: a life cycle assessment
Source: Front Digit Health. 2026 May 28;8:1788634. doi: 10.3389/fdgth.2026.1788634 (PMC13254170; doi:10.3389/fdgth.2026.1788634)
Supplement: Supplementary file 2 [file Datasheet2.docx]

S2 Appendix: Life cycle inventory and allocation approach

## Reference flows

An exhaustive list of all products and processes included in the analyses (reference flows) is displayed in Tables S1-12.

### Medical

#### Medicines

To analyze the impacts of added or avoided medicines, we classified them by class (antibiotics, antimalarials, vitamins and supplements, etc.) and by pharmaceutical form (tablet, syrup, ointment, intravenous, etc.). To simplify the analysis, we then chose the most frequently used medicine to represent each category. For tablets and capsule medicines, the number of prescriptions per year was multiplied by the number of tablets in a standard prescription to obtain the number of tablets or capsules per year. Medicines for which the prescription rate changed by less than 1% following the implementation of the Dynamic project were not included. The inventory included the amount of each product (e.g., number of tablets of a given medicine), its galenic form, its excipients composition and weight, its Active Pharmaceutical Ingredients (API) concentration, its packaging composition and weight. The packaging was either weighed or estimated. The assumptions on the composition of the packaging are detailed in the last column. The weight of the excipients was deducted from the total weight by subtracting the weight of the packaging and the active pharmaceutical ingredients (API).

| Category | Product | Type | Amount per year | API weight per unit  (mg) | Excipient weight per unit  (mg) | Packaging weight per unit  (mg) | Comment |
| --- | --- | --- | --- | --- | --- | --- | --- |
| Added | | | | | | | |
| Supplements | Iron & folate | Tablet in carton box (100 tablets per box) | 133’758 tablets | 200 | 50 | 250 | Assuming 50% of packaging weight as aluminum and 50% as carton board |
| Rehydration complement | Zinc sulfate | Tablet in carton box (10 tablets per box) | 7’916 tablets | 20 | 34 | 546 | Assuming 50% of packaging weight as aluminum and 50% as carton board |
| Deworming | Albendazole | Tablet in carton box | 8’280 tablets | 400 | 680 | 300* | Assuming 50% of packaging weight as aluminum and 50% as carton board. *Weight assumed |
| Anti-malarials | Artemether lumefantrine | Tablet in carton box (360 tablets per box) | 6’005 tablets | 140 | 238 | 622 | Assuming 50% of packaging weight as aluminum and 50% as carton board |
| Topical corticosteroids | Hydrocortisone cream | Cream in tube | 1'310 tubes | 280 | 2772 | 10’000 | Assuming 100% of packaging weight as aluminum |
| Analgesics | Paracetamol | Tablet in carton box (100 tablets per box) | 23’476 tablets | 500 | 200 | 300 | Assuming 50% of packaging weight as aluminum and 50% as carton board *weight assumed |
| Topical anti-scabies | Benzylbenzoate | Emulsion in 100ml bottle and carton box | 1'310 bottles | 28000 | 75000 | 100000 | Assuming 90% of packaging weight as glass, 10% as carton box. *Weight assumed 130g per bottle |
| Vitamins | Vitamin A | Capsules | 3'567capsules | 120 | 50 | 200 | Assuming 100% of packaging weight as maize starch 15mg of beta carotene = 25,000 IU of Vitamin A activity. |
| Antibiotic drops | Chloramphenicol eye drops | Emulsion in 5ml plastic bottle | 1’019 bottles | 25 | 4975 | 6000 | Assuming 50% of packacking weight as plastic and 50% as carton board |
| Avoided | | | | | | | |
| Cough syrups | Cough syrups | Syrup in 100ml bottle and carton box | 11’301 bottles | 100 | 99900 | 100000 | Assuming 90% of packaging weight as glass, 10% as carton box. |
| Topical antifungal | Clotrimoxazole cream | Cream in tube | 1'356 tubes | 200 | 19800 | 10000 | Assuming 100% of packaging weight as aluminum |
| Antihistamine | Cetirizine | Tablet in carton box (24 tablets per box) | 18'562 tablets | 10 | 18 | 143 | Assuming 50% of packaging weight as aluminum and 50% as carton board |
| Antibiotics | Amoxicilline | Tablet in carton box (100 tablets per box) | 735’761 tablets | 250 | 250 | 320 | Assuming 50% of packaging weight as aluminum and 50% as carton board |

*Table S1: reference flows for medicines added and avoided and assumptions on packaging composition.*

| Galenic formulation | Excipient composition |
| --- | --- |
| Tablets | 25% maize starch, 75% chemicals organics |
| Capsules | 100% chemicals organics |
| Creams | 50% distilled water, 25% paraffin, 25% chemicals organics |
| Syrup | 80% distilled water, 20% maltitolum liquidum (maize starch used as proxy) |
| Emulsion | 80% distilled water, 20% maltitolum liquidum (maize starch used as proxy) |

*Table S2: Assumptions on excipients composition*

|  | Reference flow | Ecoinvent variable |
| --- | --- | --- |
| API | Chemical organics | Chemical organics |
| Excipients | Chemical organics | Chemical organics |
|  | maize starch | maize starch |
|  | paraffin | paraffin |
|  | Distilled water | water, deionised |
| Packaging | Carton board | solid bleached and unbleached board carton |
|  | Plastic (for tablet blisters) | polyvinylchloride, emulsion polymerised |
|  |  | thermoforming of plastic sheets |
|  | aluminium | aluminium, primary, liquid |
|  | glass | packaging glass, white |
| Transport | Boat transport (default scenario) | transport, freight, sea, container ship |
|  | Air transport (sensitivity analysis) | transport, freight, aircraft, unspecified |

Table S3: ecoinvent variables used for medicines (purchase life cycle stage)

#### Tests

| Type of test | Model / Reference | Unit | Amount |
| --- | --- | --- | --- |
| Pulse oximetry | Lifebox Pulse Oximeter – Model No. AH-M1 | Number of pulse oximeters bought for the project | 44 |
|  |  | Weight (g) per pulse oximeter | 987 |
| CRP point-of-care tests | Actim CRP, 20 tests, product code 31031 ETAC(1) | Number of additional tests / year | 20610 |
|  |  | Weight (g) per test, packaging included | 9 |
| Hemoglobin point-of-care tests | Hemocue HB 201+ (2) | Number of Hemocue Analyzer bought for the project | 11 |
|  |  | Weight (g) of 1 Hemocue Analyzer | 751 |
|  |  | Number of additional tests / year | 22570 |
|  |  | Weight (g) per disposable test kit (Cuvettes), packaging included | 1.94 |
| Malaria point-of-care tests | Malaria P.f/Pan Ag Test  WHO reference number: PQDx 0347-117-00 (3) | Number of additional tests / year | 6624 |
|  |  | Weight (g) per test, packaging included | 10 |
| HIV point-of-care tests | Bioline HIV ½ 3.0  WHO reference number : PQDx 0027-012-00 (4) | Number of additional tests/year | 1492 |
|  |  | Weight (g) per test, packaging included | 18 |
| Urine tests | Urit 11 V | Number of additional tests/year | 4805 |
|  |  | Weight (g) per test, packaging included | 0.73 |
|  |  | Number of additional plastic containers to perform urine tests/year | 4805 |
|  |  | Weight (g) per plastic container | 10 |
| Supplementary material required to perform additional tests |  | Number of additional kits / year (one kits contains a pair of gloves, one lancet, one plaster and one gauze pad) | 8881 |
|  | Gloves – MSD Super Care Latex powdered examination gloves | Weight (g) per pair of gloves | 9 |
|  | Lancets – Medco Blood Lancet 25KGY | Weight (g) per lancet | 0.63 |
|  | Plaster | Weight (g) per plaster | 0.12 |
|  | Gauze pad | Weight (g) per gauze pad | 1.13 |

*Table S4: additional point-of-care tests and material required to perfom tests. Composition of CRP, HIV, Hemoglobin cuvettes and malaria tests was considered to be 80% plastic (HDPE) and 20% carton board. Composition of Urine tests was assumed to be 50% PET and 50% paper, plastic containers to perform urine tests were assumed to be 100% PET.*

|  | Reference flow | Ecoinvent variable |
| --- | --- | --- |
| CRP, HIV, Hemoglobin cuvettes and malaria tests | Plastic (HDPE) | polyethylene, high density, granulate |
|  |  | extrusion of plastic sheets and thermoforming, inline |
|  | Carton board | solid bleached and unbleached board carton |
| Urine tests | PET | polyethylene terephthalate, granulate, amorphous |
|  |  | injection moulding |
|  | Paper (strips in urine tests) | paper, woodfree, uncoated |
| Hemocue analyzers | Hemocue analyzers | consumer electronics, mobile device, smartphone |
| Pulse oximeters | Pulse oximeters | battery, Li-ion, NMC111, rechargeable, prismatic |
|  |  | cable, network cable, category 5, without plugs |
|  |  | liquid crystal display, unmounted, mobile device |
|  |  | plug, inlet and outlet, for computer cable |
|  |  | polypropylene, granulate |
|  |  | printed wiring board, mounted mainboard, laptop computer, Pb free |
| Transport | Air transport to Tanzania (16’000km) | transport, freight, aircraft, unspecified |

Table S5: ecoinvent variables used for tests (purchase life cycle stage)

### Digital

| Item | Amount | Weight per unit (g) | Ecoinvent variable |
| --- | --- | --- | --- |
| Tablets | 51 | 392 | consumer electronics, mobile device, tablet |
| Portable computers | 2 | 1614 | computer, laptop |
| Rasperry Pi | 42 | 91 | electronic component, passive, unspecified |
| Routers | 42 | 333 | electronic component, passive, unspecified |
| Security box to store routers (steel) | 40 | 5000 | metal working, average for steel product manufacturing |
|  |  |  | steel, low-alloyed |
| UPS | 23 | 4170 | battery, Li-ion, rechargeable, prismatic |
| Solar panels | 1.2 m^2^ |  | photovoltaic panel, ribbon-Si |
| Solar batteries | 3 | 22200 | battery, Li-ion, rechargeable, prismatic |
| Ecoflow batteries | 11 |  | battery, Li-ion, rechargeable, prismatic |

Table S6: Reference flows included in the digital category

| Item | Energy use (kWh/year) | Assumptions | Sources |
| --- | --- | --- | --- |
| 1 tablet | 11.9 | Based on charging it every other day | Sust-it (5) |
| 51 tablets (49 in Health facilities + 2 at District level) | 606.9 |  |  |
| 1 laptop (at central level, Dar es Salaam) | 37.8 | 8 hours per day, 249 days per year | ecoinvent |
| 2 laptops (at Tanzanian district level) | 75.7 |  |  |
| 1 server (32 GB) | 1760.3 | Used 24/7 | [GO Climate](https://www.goclimate.com/blog/the-carbon-footprint-of-servers/) (6) |
| 17.29 GB stored on servers (at central level, Dar es Salaam) | 951.0 |  |  |
| 1.089 GB stored on servers (at Unisanté, Switzerland) | 59.9 |  |  |

*Table S7: Assumptions on the power consumption of the different electronic tools*

| Location | Electricity mix |
| --- | --- |
| Health facilities | 85% grid mix Tanzania; 15% photovoltaic |
| District – Mbeya & Ifakara, Tanzania | 100% grid mix Tanzania |
| Central – Dar es Salaam, Tanzania | 100% grid mix Tanzania |
| Unisanté – Lausanne, Switzerland | 100% grid mix Switzerland |

*Table S8: Assumptions on electricity mix by location*

| Power source | Production % |
| --- | --- |
| Hydro, reservoir | 33% |
| Natural gas | 48% |
| Oil | 18% |
| Wood chips, heat and power cogeneration | 1% |

*Table S9: Tanzanian main grid mix* (7)

### Logistics

| Item | Unit | Amount | Ecoinvent variable |
| --- | --- | --- | --- |
| Vehicles used for supervision visits | Toyota Land cruisers |  | transport, passenger car, large size, diesel, EURO 3 |
| Distance travelled for project implementation (only for 1^st^ year of project) | Kilometers | 8000 |  |
| Distance travelled for supervision visits / year | Kilometers | 8000 |  |
| Vehicles used by clinicians to attend centralized training | Bus |  | transport, regular bus |
| Number of clinicians attending centralized training once a year | People | 100 |  |
| Average distance travelled by clinicians to attend centralized training | Kilometers | 80 |  |

*Table S10: Reference flows included in the “logistics” category*

### Waste (end of life)

| Item | Amount (kg/year) | Mixed plastics | Paper & board | Aluminium | Glass | Electrical components |
| --- | --- | --- | --- | --- | --- | --- |
| Electric and electronic devices (Health facilities) | 46.3 | 50% | 0% | 0% | 10% | 40% |
| Electric and electronic devices (District) | 1.98 | 50% | 0% | 0% | 10% | 40% |
| Single use medical equipment | 679.5 | 80% | 20% | 0% | 0% | 0% |
| Medicine packaging - administered | 115.2 | 13% | 27% | 31% | 28% | 0% |
| Medicine packaging - avoided | 554.6 | 9% | 23% | 18% | 50% | 0% |

*Table S11: waste quantities and composition*

| Waste management mode | Mixed plastics | Paper & board | Aluminium | Glass | Electrical components |
| --- | --- | --- | --- | --- | --- |
| Disposed in sanitary landfill |  |  | 100% |  |  |
| Disposed in unsanitary landfill | 50% | 50% |  | 50% |  |
| Disposed in open dump | 50% | 50% |  | 50% |  |
| Open burning |  |  |  |  | 100% |

*Table S12: Waste management for different categories of material*

## Allocation approach

| Purchased good | Category | Description | Allocation factor |
| --- | --- | --- | --- |
| Tablets (iPads) | Digital | Tablets used for medAL-Reader et medAL-Monitor. Expected life duration: 3 years | 1/3 = 33% |
| Laptop computers | Dgital | Laptop model XX used for medAL-outbreak & medAl-Monitor supervision. Expected life duration: 5 years | 1/5 = 20% |
| Raspberry Pi | Digital | Electronic device Expected life duration: 5 years | 1/5 = 20% |
| Routers | Digital | Electronic device Expected life duration: 5 years | 1/5 = 20% |
| UPS batteries | Digital | Uninterruptible power supply. Expected life duration: 5 years. | 1/5 = 20% |
| Solar batteries | Digital | Assumption: Li-ion rechargeable battery Expected life duration: 10 years | 1/10 = 10% |
| Ecoflow batteries | Digital | Assumption: Li-ion rechargeable battery Expected life duration: 10 years | 1/10 = 10% |
| Solar panels | Digital | Photovoltaic panels, technology unspecified. Expected life duration: 15 years | 1/15 = 6.7% |
| Security box | Digital | Steel boxes for tablet storage. Expected life duration: 10 years | 1/10 = 10% |
| Pulse oxiometers | Medical | Lifebox Pulse Oximeter - Model No. AH-M1. Expected life duration: 10 years | 1/10 = 10% |
| CRP tests | Medical | Actim rapid tests CRP. Single use | 100% |
| HB tests | Medical | Hemoglobin tests. Single use | 100% |
| HemoCue analysers | Medical | HemoCue analyzer used in combination to HB tests. Expected life duration: 10 years | 1/10 = 10% |
| Urine tests | Medical | Urine tests (paper strips) with single use plastic container. | 100% |
| HIV rapid tests | Medical | HIV rapid tests. Single use. | 100% |
| Malaria rapid tests | Medical | Malaria rapid tests. Single use. | 100% |
| Supplementary equipment kit for rapid tests | Medical | 1 pair of gloves, lancet for sampling, disinfectant, compress and plaster. Single use | 100% |

*Table S13: allocation factors for digital and medical equipment and devices. Allocation was applied to purchased digital and medical equipment in consideration of their lifespan, when expected to be longer than one year.*

References

1. Actim® CRP [Internet]. Actim. [cited 2025 Jun 11]. Available from: https://www.actimtest.com/actim-crp/

2. Test de l’hémoglobine - HemoCue Hb 201+ System [Internet]. [cited 2025 Jun 11]. Available from: https://www.hemocue.fr/fr-fr/solutions/h%C3%A9matologie-/hemocue-hb-201plus-system

3. SD BIOSENSOR | PRODUCTS [Internet]. [cited 2025 Jun 11]. Available from: https://www.sdbiosensor.com/product/product_view?product_no=213

4. PQDx_0027-012-00_BiolineHIV_1-2-3_v5.pdf [Internet]. [cited 2025 Jun 11]. Available from: https://extranet.who.int/prequal/sites/default/files/whopr_files/PQDx_0027-012-00_BiolineHIV_1-2-3_v5.pdf

5. Lammas R. Sust-it. 2012 [cited 2023 Mar 9]. How much electricity does an iPad tablet computer use? Available from: https://www.sust-it.net/blog/how-much-electricity-does-an-ipad-tablet-computer-use/

6. kalle. The Carbon Footprint of Servers - GoClimate Blog [Internet]. 2019 [cited 2023 Mar 9]. Available from: https://www.goclimate.com/blog/the-carbon-footprint-of-servers/

7. IEA [Internet]. [cited 2023 Mar 9]. World Energy Outlook 2019 – Analysis. Available from: https://www.iea.org/reports/world-energy-outlook-2019
